# The Frequency of Langerhans Islets *β*-Cells Autoantibodies (Anti-GAD) in Georgian Children and Adolescents with Chronic Autoimmune Thyroiditis

**DOI:** 10.1155/2016/6597091

**Published:** 2016-06-27

**Authors:** Mariam Balakhadze, Elene Giorgadze, Marina Lomidze

**Affiliations:** ^1^Ivane Javakhishvili Tbilisi State University, 0179 Tbilisi, Georgia; ^2^V. Iverieli Endocrinology Metabology Dietology Center “ENMEDIC”, 9 Tsinandali Street, 0144 Tbilisi, Georgia; ^3^National Institute of Endocrinology, 2/6 Lubliana Street, 0159 Tbilisi, Georgia

## Abstract

*Aim*. Chronic autoimmune thyroiditis and type 1 diabetes mellitus are organ-specific autoimmune diseases. There is large evidence that autoimmunity against the thyroid gland in patients with type 1 diabetes mellitus is increased, but little is known about anti-islet cell autoimmune status in patients with chronic autoimmune thyroiditis. We evaluated the concentration of antibodies against glutamic acid decarboxylase (GAD) which are widely used as a diagnostic and predictive tool for type 1 diabetes mellitus, in school-aged Georgian children with chronic autoimmune thyroiditis.* Methods*. The frequency of anti-GAD antibodies was measured in Georgian school-aged children with chronic autoimmune thyroiditis and compared to healthy age and sex matched controls.* Results*. Of the 41 patients with chronic autoimmune thyroiditis 4 (9.8%) were positive for GAD antibodies. The frequency of GAD positivity in the chronic autoimmune thyroiditis group was significantly higher than in the control subjects (*P* = 0.036).* Conclusion*. In the study we found that the frequency of GAD antibody positivity in autoimmune thyroiditis patients was significantly higher (9.8%, *P* = 0.036) than in the control group. Our findings support the concept that patients with autoimmune thyroid disease may develop type 1 diabetes mellitus in future life.

## 1. Introduction

Hashimoto's thyroiditis or chronic autoimmune thyroiditis is the most common cause of acquired hypothyroidism or goiter in children over 6 years of age in iodine-sufficient areas of the world. The patients generally are genetically predisposed to the disease and family history of thyroid disorder is seen in up to 30–40% of patients [1, 2]. This condition is characterized by lymphocytic infiltration and destruction of thyroid parenchyma and high serum concentration of autoantibodies against thyroid tissue. Documenting high serum concentrations of anti-thyroid peroxidase (anti-TPO) and/or anti-thyroglobulin (anti-TG) autoantibodies is sufficient to diagnose chronic autoimmune thyroiditis as the cause of a patient's hypothyroidism [3]. The prevalence of autoimmune thyroiditis (defined by characteristic echographic pattern and elevated concentration of anti-TPO and/or anti-TG autoantibodies) in schoolchildren is approximately 2.5% [4].

The cause of thyroid autoimmunity is a combination of genetic and environmental factors. The genetic factors which are associated with Hashimoto's thyroiditis lie in the human leukocyte antigen (HLA) complex, while an association with cytotoxic T-lymphocyte-associated protein 4 (CTLA-4) or a closely linked gene has also been described [5, 6].

Type 1 diabetes mellitus, like autoimmune thyroiditis, is an organ-specific autoimmune disease which is caused by a complex interaction of genetic and environmental factors. It is reported that HLA and CTLA-4 polymorphism is associated with type 1 diabetes mellitus as well as with autoimmune thyroid disease [4, 5, 7].

Most patients with newly diagnosed type 1 diabetes mellitus have elevated concentrations of autoantibodies against Langerhans islet *β*-cells. The antigens for these antibodies are insulin (IAA), glutamic acid decarboxylase (GAD), insulinoma associated autoantigen 2 (IA2A), and zinc transporter 8 (ZnT8A). The autoantibodies appear in the blood at as early as 6 months of age and are detectable several years before the clinical manifestation of the disease [8, 9]. These antibodies can have both a diagnostic and predictive value for type 1 diabetes mellitus.

The incidence of type 1 diabetes has been increasing worldwide for several decades [10]. If the incidence rate continues to increase on the existing path, there is the risk of doubling of global incidence [7].

There is large evidence that autoimmunity against the thyroid gland in patients with type 1 diabetes mellitus is increased by up to 20–30% [11, 12]. In contrast, little is known about anti-islet cell autoimmune status in patients with autoimmune thyroid disease. Therefore we evaluated the concentration of antibodies against glutamic acid decarboxylase (GAD) which are widely used as a diagnostic and predictive tool for type 1 diabetes mellitus.

## 2. Design and Methods

### 2.1. Subjects

We studied Georgian school-aged children, up to 16 years of age, who had been diagnosed as having chronic autoimmune thyroiditis. The patients were recruited from the V. Iverieli Endocrinology Metabology Dietology Center “ENMEDIC,” a clinic which has nearly a hundred years of experience in the field of endocrinology and to which patients with endocrine pathology are referred from all regions of Georgia. The exclusion criteria for this study were diabetes mellitus, first-degree relatives with type 1 diabetes mellitus, other autoimmune conditions, Turner Syndrome, and Down Syndrome. The diagnosis of chronic autoimmune thyroiditis was established by the responsible pediatric endocrinologist, confirmed by elevated levels of serum thyrotropin (TSH) and autoantibodies against thyroid peroxidase and/or thyroglobulin. The control group was chosen from randomly selected schools in different regions of Georgia. The exclusion criteria for control subjects were first-degree relatives with type 1 diabetes mellitus and a history of thyroid disease. The serum concentrations of anti-TPO and anti-TG antibodies were investigated in the control group and were not elevated, within reference range, in all subjects recruited in this group. Positivity of GAD antibodies was compared in two groups.

### 2.2. Assays

Glutamic acid decarboxylase antibodies were detected using quantitative ELISA (produced by RSR Limited, UK). The assay has specificity of 99% and sensitivity of 72%. Cut-off value for positive results was established as ≥5 U/mL. To assess the plasma concentration of anti-TPO and anti-TG antibodies we used the ADVIA Centaur XP anti-TPO and anti-TG assays (produced by Siemens) using chemiluminescent technology. Samples were defined as anti-TPO positive when the level was higher than 60 IU/mL and anti-TG positive when the level was higher than 60 IU/mL. We use ADVIA Centaur XP TSH assay (produced by Siemens) for measurement of TSH plasma concentration, and reference range was 04–4.0 *μ*IU/mL, and we use ADVIA Centaur XP FT4 assay (produced by Siemens) for measurement of free thyroxine (FT4) plasma concentration, and reference range was 0.8–2.0 ng/dL.

### 2.3. Statistical Analyses

Fisher's exact probability test was used to assess the significance of differences of GAD positivity between the patients with autoimmune thyroiditis and healthy control subjects. Statistical significance was defined as *P* < 0.05.

### 2.4. Ethical Aspects

The study was approved by the Local Ethical Committee of V. Iverieli Endocrinology Metabology Dietology Center “ENMEDIC,” study number ENM-001, in agreement with Helsinki Declaration (1977) regarding ethical standards for clinical studies in medicine. Informed consent was obtained from the children and/or from their legal guardians.

## 3. Results 

The patient group consisted of 41 children (10 males and 31 females), aged from 6 to 16 (mean 13.15; median 13.00; SD 2.231). As the control group there were 51 healthy subjects (17 males and 34 females), age from 6 to 16 years (mean 12.96; median 13.00; SD 2.441).

In the study group 37 of the 41 patients (90.2%) were receiving treatment with levothyroxine, three (7.3%) had primary hypothyroidism (without goiter) with TSH elevation (TSH values were from 11.57 to 137.42 *μ*IU/mL; median: 74.89), six (14.6%) had subclinical hypothyroidism (without goiter) (TSH values were from 5.12 to 7.36 *μ*IU/mL; median: 5.28), five (12.2%) had primary hypothyroidism with diffuse goiter (TSH values were from 15.24 to 150.01 *μ*IU/mL; median: 37.45), 21 (51.2%) had subclinical hypothyroidism with diffuse goiter (TSH values were from 4.43 to 6.28 *μ*IU/mL; median: 4.98), four (9.8%) had subclinical hypothyroidism with nodular goiter (TSH values were from 4.67 to 4.99 *μ*IU/mL; median: 4.88), one had primary hypothyroidism with nodular goiter, and one presented with hyperthyroidism and diffuse goiter (Table 1).

Of the 41 patients with autoimmune thyroiditis 4 (9.8%) were positive for GAD antibodies and their details are shown in Table 2. None of the 51 control subjects was positive for GAD.

In the four GAD positive patients three had diffuse goiter and one had nodular goiter, and among them two had primary hypothyroidism and two had subclinical hypothyroidism (Table 2).

The frequency of GAD positivity in the autoimmune thyroiditis group was significantly higher than in the control subjects (*P* = 0.036) (Table 3).

So far the GAD positive patients are under the close observation. We plan to reevaluate the patients annually. The assessment of other anti-islet antibodies, antibodies against adrenal glands, and IgA tissue transglutaminase antibodies is also planned.

## 4. Discussion

Glutamic acid decarboxylase of 65 kDa (GAD65) is one of the major autoantigens for type 1 diabetes mellitus. Glutamic acid decarboxylase catalyzes the synthesis of gamma-aminobutyric acid (GABA), which is known as a major inhibitory neurotransmitter in the central nervous system (CNS), but it is also present outside the CNS. It is also found in *β*-cells of islets of Langerhans, but its functional role remains largely unknown. Antibodies to glutamic acid decarboxylase serve as a predictor and marker of type 1 diabetes mellitus [13]. The predictive sensibility of GAD antibodies for type 1 diabetes mellitus in the general population is approximately 50% [14]. In our study we show that the frequency of GAD positivity in the autoimmune thyroiditis group was significantly higher than in the control subjects (*P* = 0.036). Our findings support the concept that patients with autoimmune thyroid disease may be at high risk of developing type 1 diabetes mellitus, as is concluded by the studies of healthy children from the general population, which show marked increase of incidence of type 1 diabetes mellitus in subjects with positive serum antibodies against pancreatic islet cells [15, 16].

According to our findings we concluded that the screening for GAD antibodies in patients with autoimmune thyroid disease may reveal the individuals with increased risk of developing type 1 diabetes mellitus; however, the importance of screening autoantibodies and then follow-up of antibody positive patients remains controversial, as the disease cannot be prevented so far [17]. But it is still very important to reveal the different risk groups as recent studies revealed a markedly reduced rate of diabetic ketoacidosis and other complications at onset of type 1 diabetes in individuals screened for islet autoantibodies [18].

## 5. Conclusion

In the study we found that the frequency of GAD antibody positivity in autoimmune thyroiditis patients was significantly higher (9.8%, *P* = 0.036) than in the control group. Our findings support the concept that patients with autoimmune thyroid disease may develop type 1 diabetes mellitus in future life.

Though the onset of type 1 diabetes cannot be prevented, it is still very important to reveal the different risk groups as the knowledge of the increased risk can help to prevent acute onset of the disease with ketoacidosis and concomitant morbidities.

## Figures and Tables

**Table 1:** Clinical characteristics of 41 patients aged 6 to 16 with autoimmune thyroiditis and the control group.

	Total *N*	Females/males	TSH *µ*IU/mL median (range)	Anti-TPO IU/mL median (range)	Anti-TG IU/mL median (range)
Primary hypothyroidism (without goiter)	3	3/0	74.89 (11.57–137.42)	156.9 (153.13–318.05)	342.69 (217.01–375.21)
Subclinical hypothyroidism (without goiter)	6	4/2	5.28 (5.12–7.36)	239.59 (78.07–569.56)	379.84 (82.7–482.35)
Primary hypothyroidism with diffuse goiter	5	4/1	37.45 (15.24–150.01)	480.27 (206–653.38)	—
Subclinical hypothyroidism with diffuse goiter	21	15/6	4.98 (4.43–6.28)	211.57 (72.95–517.32)	292.19 (136.47–546.24)
Primary hypothyroidism with nodular goiter	1	1/0	94.78	517.32	—
Subclinical hypothyroidism with nodular goiter	4	3/1	4.88 (4.67–4.99)	243.98 (145.22–526.75)	619.53 (214.15–1024.91)
Diffuse goiter with hyperthyroidism	1	1/0	0.01	238.78	—
Levothyroxine treatment^*∗*^	37	27/10	5.46 (4.43–150.01)	216.35 (72.95–653.38)	333.42 (82.7–1024.91)
All patients	41	31/10	5.26 (0.01–150.01)	236.35 (72.95–653.38)	333.42 (82.7–1024.91)
Control group	51	34/17	1.84 (0.53–3.78)	36.91 (25.02–51.35)	39.85 (29.76–52.06)

^*∗*^Including all patients from all subgroups which receive levothyroxine treatment.

**Table 2:** Clinical characteristics of GAD positive patients.

	Patient 1	Patient 2	Patient 3	Patient 4
Sex	Female	Female	Female	Male
Age	11	14	12	13
FT4 titre pmol/L (first visit)		12.61	11.33	13.64
TSH titre *µ*IU/mL (first visit)	94.78	5.12	15.24	4.43
Anti-TPO titre IU/mL	517.32	327.25	480.27	95
Anti-TG titre IU/mL		433.96		
Anti-GAD titre U/mL	231.01	413.79	5.92	23.56
Type of goiter	Nodular goiter	Diffuse goiter	Diffuse goiter	Diffuse goiter
Current dose of LT4 *µ*g/day	100	125	112.5	100

**Table 3:** Frequency of GAD antibodies in patients with autoimmune thyroiditis and the control subjects.

	Patients with autoimmune thyroiditis	Control subjects	*P*
*n*	41	51	
Age (years)	13.39 ± 1.96	12.98 ± 2.39	
Sex (male/female)	10/31	17/34	
Anti-GAD positive (*n*/%)	4/9.8	0	0.036

## References

[1] Hollowell J. G., Staehling N. W., Flanders W. D. (2002). Serum TSH, T4, and thyroid antibodies in the United States population (1988 to 1994): National Health and Nutrition Examination Survey (NHANES III). *Journal of Clinical Endocrinology and Metabolism*.

[2] Fisher D. A., Grueters A., Sperling M. (2008). Thyroid disorders in childhood and adolescence. *Pediatric Endocrinology*.

[3] Huang S. A., Kappy M. S., Allen D. B., Geffner M. E. (2009). Thyroid. *Pediatric Practice, Endocrinology*.

[4] Kaloumenou I., Mastorakos G., Alevizaki M. (2008). Thyroid autoimmunity in schoolchildren in an area with long-standing iodine sufficiency: correlation with gender, pubertal stage, and maternal thyroid autoimmunity. *Thyroid*.

[5] Weetman A. P. (2003). Autoimmune thyroid disease: propagation and progression. *European Journal of Endocrinology*.

[6] Chistiakov D. A., Turakulov R. I. (2003). CTLA-4 and its role in autoimmune thyroid disease. *Journal of Molecular Endocrinology*.

[7] Atkinson M. A., Eisenbarth G. S., Michels A. W. (2014). Type 1 diabetes. *The Lancet*.

[8] Bingley P. J. (2010). Clinical applications of diabetes antibody testing. *Journal of Clinical Endocrinology and Metabolism*.

[9] Hummel M., Bonifacio E., Schmid S., Walter M., Knopff A., Ziegler A.-G. (2004). Brief communication: early appearance of islet autoantibodies predicts childhood type 1 diabetes in offspring of diabetic parents. *Annals of Internal Medicine*.

[10] Dabelea D. (2009). The accelerating epidemic of childhood diabetes. *The Lancet*.

[11] Kordonouri O., Klinghammer A., Lang E. B., Grüters-Kieslich A., Grabert M., Holl R. W. (2002). Thyroid autoimmunity in children and adolescents with type 1 diabetes: a multicenter survey. *Diabetes Care*.

[12] Hansen D., Bennedbæk F. N., Høier-Madsen M., Hegedüs L., Jacobsen B. B. (2003). A prospective study of thyroid function, morphology and autoimmunity in young patients with type 1 diabetes. *European Journal of Endocrinology*.

[13] Siljander H. T., Veijola R., Reunanen A., Virtanen S. M., Åkerblom H. K., Knip M. (2007). Prediction of type 1 diabetes among siblings of affected children and in the general population. *Diabetologia*.

[14] Sørgjerd E. P., Thorsby P. M., Torjesen P. A., Skorpen F., Kvaløy K., Grill V. (2015). Presence of anti-GAD in a non-diabetic population of adults; time dynamics and clinical influence: results from the HUNT study. *BMJ Open Diabetes Research & Care*.

[15] Locatelli M., Songini M., Bottazzo G. F. (1999). IDDM-Sardinia Project: a study model on the etiopathogenesis of insulin-dependent diabetes mellitus and other autoimmune pathologies. Gruppi di studio IDDM-Sardegna. *Annali dell'Istituto Superiore di Sanità*.

[16] Schlosser M., Strebelow M., Wassmuth R. (2002). The Karlsburg type 1 diabetes risk study of a normal schoolchild population: association of *β*-cell autoantibodies and human leukocyte antigen-DQB1 alleles in antibody-positive individuals. *Journal of Clinical Endocrinology and Metabolism*.

[17] Thrower S. L., Bingley P. J. (2009). Strategies to prevent type 1 diabetes. *Diabetes, Obesity and Metabolism*.

[18] Winkler C., Schober E., Ziegler A.-G., Holl R. W. (2012). Markedly reduced rate of diabetic ketoacidosis at onset of type 1 diabetes in relatives screened for islet autoantibodies.. *Pediatric diabetes*.

